# Interdisciplinary Profile: An Established Chemist Journeys into Different Disciplines

**DOI:** 10.1016/j.isci.2020.101088

**Published:** 2020-05-01

**Authors:** James R. Heath

**Affiliations:** 1 Institute for Systems Biology, Seattle, WA, USA

**Figure:**
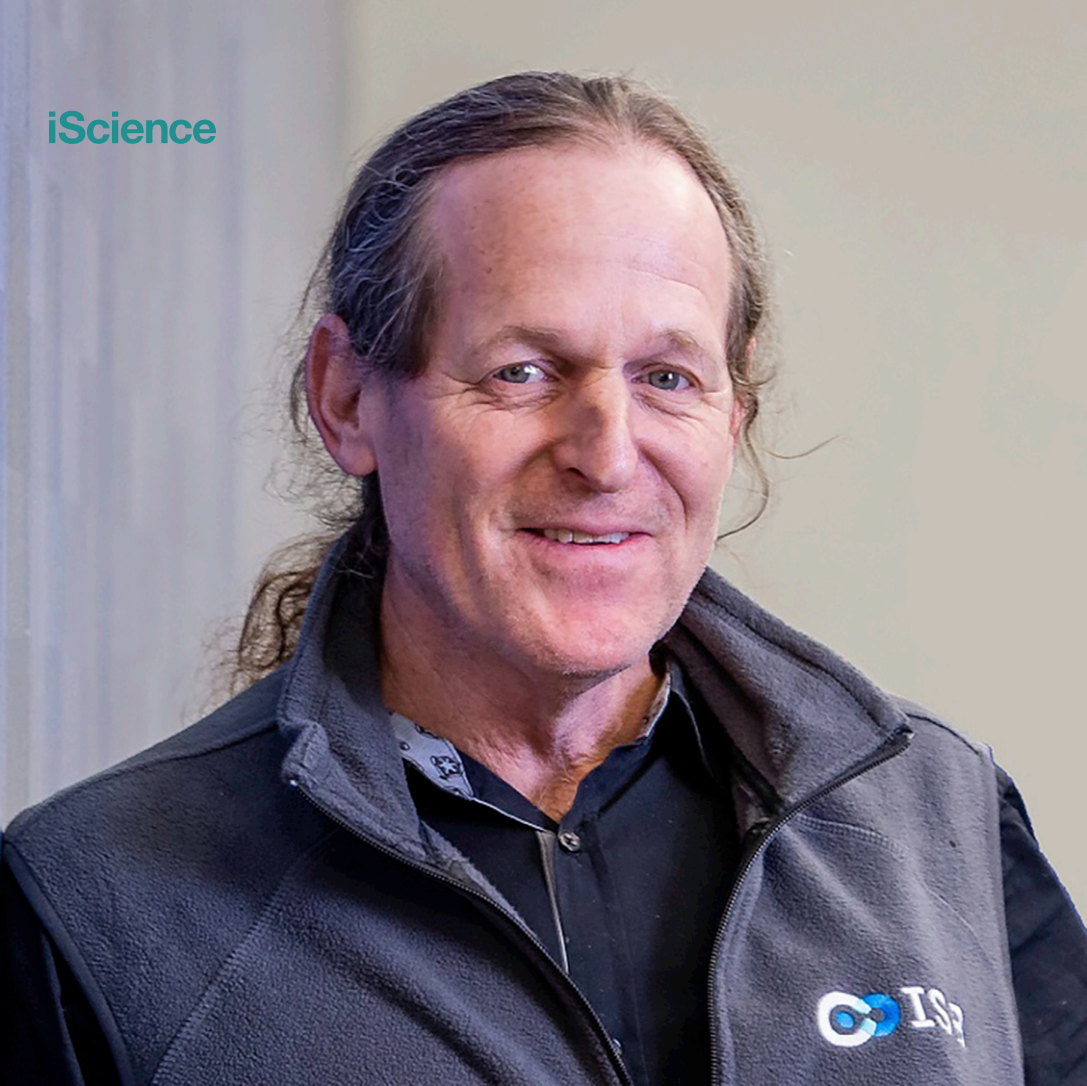

Interdisciplinary research projects are often encouraged by institutions, funding bodies, and journals, yet the inspiration to conduct such a project first truly originates with individuals. One such person, Dr. James R. Heath, who has been a robust contributor to the field of chemical physics for the last two decades, now pursues a wide breadth of projects based on his ever-evolving interests and finds himself crossing multiple disciplinary borders along the way.

Heath is a graduate of Rice University, where he was a junior collaborator in the discovery of C60 and the fullerenes, which resulted in three senior members of this group receiving the Nobel Prize in Chemistry in 1996. Currently, he is the President and Professor at the Institute for Systems Biology in Seattle, as well as Professor of Molecular and Medical Pharmacology at UCLA. Below, he shares some of the difficulties he has experienced in his venture into new research areas and ensures that there is a wealth of knowledge to be discovered at these multidisciplinary intersections.

## Proximity

### Crossing Borders in Science Is a Way to Find Solutions to Complex Problems

Many of the most exciting scientific problems are not neatly defined by one discipline or another.

Many of the most exciting scientific problems are not neatly defined by one discipline or another. I come from a field (chemical physics) that is extremely quantitative in both the science of measurement and the underlying discipline of the physico-chemical laws. These are traits that I love and that I am constantly wanting to bring into my science. However, the scientific questions that I want to address are associated with the biology of human health and disease. Thus, I end up doing cross-borders science. Of course, sometimes working across scientific disciplines can make one look naive. More often, however, I think it is an area that is rich with discovery.

## Language

### Collaboration Requires Being Both a Good Teacher and a Good Student

Most disciplines (especially biology) are dense with jargon that can mask the fundamentals of the science itself.

“Language” is one of the biggest barriers to working across disciplines. The difficulty of language highlights the need to (patiently) work with someone who has deep expertise in any new area that one ventures into. That collaborator must, in turn, also be a patient teacher and a willing student. The willingness of collaborators to be good teachers and students of each other is illustrated by an experience of mine from many years ago. When I was first learning about cancer biology, Charles Sawyers, who was then at UCLA, agreed to give me a short course, one-on-one. On one of our first lessons, we discussed mTOR for 30 min, getting nowhere. To Charles, mTOR is an important signaling protein (mammalian target of rapamycin), whereas to me, mTOR was a unit of measurement—equal to 10^−6^ atmospheres of pressure! Of course, mTOR is both things! Anyway, we finally figured out what the other was speaking about, and made progress. Such confusion is common. Most disciplines (especially biology) are dense with jargon that can mask the fundamentals of the science itself.

## Research Methods

### Formal Re-training Might Not Be Necessary

We re-train (or evolve our science) through collaborations with colleagues who can be good teachers. Science is really about solving problems and new discovery—neither of which are well taught through formal training, but only through experience. Training in a new field will involve making rookie mistakes and asking stupid questions. Don't be afraid of either one!

## Governance

### Avoid Creating a Patchwork Proposal

Nevertheless, it helps if the proposal is written so that a non-expert can understand why the proposed work is important and get the essence of the work plan.

For sure there isn't a one size fits all. I think it helps to get to know individuals at the federal agency or foundation for whom you are seeking funding support and to understand how the proposals will be evaluated. Cross-disciplinary proposals are always the toughest to get refereed. Most agencies understand that and try to accommodate that difficulty, but with varying success. Nevertheless, it helps if the proposal is written so that a non-expert can understand why the proposed work is important and get the essence of the work plan. When writing a cross-disciplinary grant, I think there is always the temptation to have those scientists with different expertise write the relevant parts of the grant. However, I find that to be a poor strategy, since it can produce a patchwork proposal, which will usually get rejected. It is almost always a better strategy to have the main PI write the proposal. Get your colleagues to give you a harsh assessment, so that the proposal isn't naive. However, preparing a proposal should be a learning (and teaching) experience.

## Publication

### Seek out Critical Feedback before Submitting

I also will present unpublished work in seminars to get critical feedback, so as to ultimately make the paper stronger.

There are lots of issues to unpack here, and I'll just touch on a few. First, just like with cross-disciplinary proposals, it can be difficult to get a cross-disciplinary paper fairly refereed. It can be useful to have colleagues with relevant expertise critique your work first. I also will present unpublished work in seminars, to get critical feedback, so as to ultimately make the paper stronger. One can also suggest to the editor that specific referees comment on specific parts, but that can be challenging. I find that parsing different parts of a single cross-disciplinary story into different papers typically does a disservice to the science. In other words, I guess I don't really have a good answer here. Some of our highest impact work took over 2 years to get published and was only marginally better when it finally was accepted relative to when it was initially submitted. Alternatively, the refereeing process can often be of great assistance to writing a good paper.

## Conclusion

### Follow Your Interests, Wherever They Lead

In the end, it is all about teaching, being a good student always, and having fun.

We have universities that are divided up into a “Germanic” structure, with teaching and degree granting given with the different departments of chemistry, physics, math, engineering, history, biology, etc. This structure was established nearly 200 or so years ago and was designed around the problems our now-distant ancestors were trying to solve. Modern science problems, whether they are related to the environment, to renewable energy, to human health, etc., don't really fit into any one of these disciplines. I urge my students and postdocs to work on what they think is interesting, at least within the context of the many projects that go on within my group. I also encourage them to seek out collaborations that can help them solve those problems. In the end, it is all about teaching, being a good student always, and having fun.

